# The bivariate NRIP1/ZEB2 RNA marker permits non-invasive presymptomatic screening of pre-eclampsia

**DOI:** 10.1038/s41598-020-79008-4

**Published:** 2020-12-14

**Authors:** Vera Manders, Allerdien Visser, Remco Keijser, Naomi Min, Ankie Poutsma, Joyce Mulders, Tarah van den Berkmortel, Marjolein Hortensius, Aldo Jongejan, Eva Pajkrt, Erik A. Sistermans, Daoud Sie, Myron G. Best, Tom Würdinger, Marjon de Boer, Gijs Afink, Cees Oudejans

**Affiliations:** 1 Department of Clinical Chemistry, Amsterdam UMC, VU University Medical Center, Amsterdam, The Netherlands; 2 Reproductive Biology Laboratory, Amsterdam UMC, Academic Medical Center, Amsterdam, The Netherlands; 3 Department of Clinical Epidemiology, Biostatistics and Bioinformatics, Amsterdam UMC, Academic Medical Center, Amsterdam, The Netherlands; 4 Department of Obstetrics/Gynecology, Amsterdam UMC, Academic Medical Center, Amsterdam, The Netherlands; 5 Department of Clinical Genetics, Amsterdam UMC, VU University Medical Center, Amsterdam, The Netherlands; 6 Department of Neurosurgery, Amsterdam UMC, VU University Medical Center, Amsterdam, The Netherlands; 7 Department of Pathology, Amsterdam UMC, VU University Medical Center, Amsterdam, The Netherlands; 8 Brain Tumor Center Amsterdam, Amsterdam UMC, VU University Medical Center, Amsterdam, The Netherlands; 9 Department of Obstetrics/Gynecology, Amsterdam UMC, VU University Medical Center, Amsterdam, The Netherlands

**Keywords:** Biomarkers, Predictive markers

## Abstract

Using genome-wide transcriptome analysis by RNA sequencing of first trimester plasma RNA, we tested whether the identification of pregnancies at risk of developing pre-eclampsia with or without preterm birth or growth restriction is possible between weeks 9–14, prior to the appearance of clinical symptoms. We implemented a metaheuristic approach in the self-learning SVM algorithm for differential gene expression analysis of normal pregnancies (n = 108), affected pregnancies (n = 34) and non-pregnant controls (n = 19). Presymptomatic candidate markers for affected pregnancies were validated by RT-qPCR in first trimester samples (n = 34) from an independent cohort. *PRKG1* was significantly downregulated in a subset of pregnancies with birth weights below the 10thpercentile as shared symptom. The *NRIP1/ZEB2* ratio was found to be upregulated in pregnancies with pre-eclampsia or trisomy 21. Complementary quantitative analysis of both the linear and circular forms of *NRIP1* permitted discrimination between pre-eclampsia and trisomy 21. Pre-eclamptic pregnancies showed an increase in linear *NRIP1* compared to circular *NRIP1*, while trisomy 21 pregnancies did not.

## Introduction

Maternal plasma RNA sequencing allows non-invasive in vivo monitoring of placental function^1–3^. When complemented with single-cell RNA sequencing of the placental and maternal cells that contribute to the pool of cell-free RNA in plasma, the cell-specific gene signatures yield a bioinformatic barcode for non-invasive diagnostics of pregnancy-associated diseases with placental insufficiency^3^. This was demonstrated using the gene signature for the extravillous trophoblast, allowing molecular confirmation of pre-eclampsia at the symptomatic stage^3^. For another prevalent pregnancy disorder, spontaneous preterm birth (SPB), this kind of approach permitted classification even before the appearance of symptoms^4^. A set of 7 cell-free RNA transcripts in maternal plasma identified after RNA sequencing allowed classification of SPB up to two months in advance of labor^4^.

It remains to be tested if these accomplishments for the assessment of fetal and maternal well-being will permit predictive screening in early pregnancy as well^5^. A prerequisite to be clinically effective for diagnostics, risk classification and therapy is to have any RNA-based screening method be applicable and informative in the first trimester. This gestational period during which the essential vascular connection between mother and fetus is established and the placenta becomes fully hemochorial, is the time window of the pathophysiological start of the majority of pregnancy-associated diseases with placental insufficiency^6^. Presymptomatic identification of women at risk will make it possible to prescribe low-dose aspirin, proven to reduce the incidence of early pre-eclampsia in a selected population. Besides presymptomatic monitoring, the design of other effective therapies might also become available when primary, yet unrecognized pathways allowing targeted novel treatments are identified as well.

We applied a metaheuristic strategy for maternal plasma RNA sequencing analysis to test if the identification of pregnancies at risk is possible prior to the presence of clinical symptoms.

## Results and discussion

### Maternal plasma RNA sequencing: sample selection

**Figure 1:**
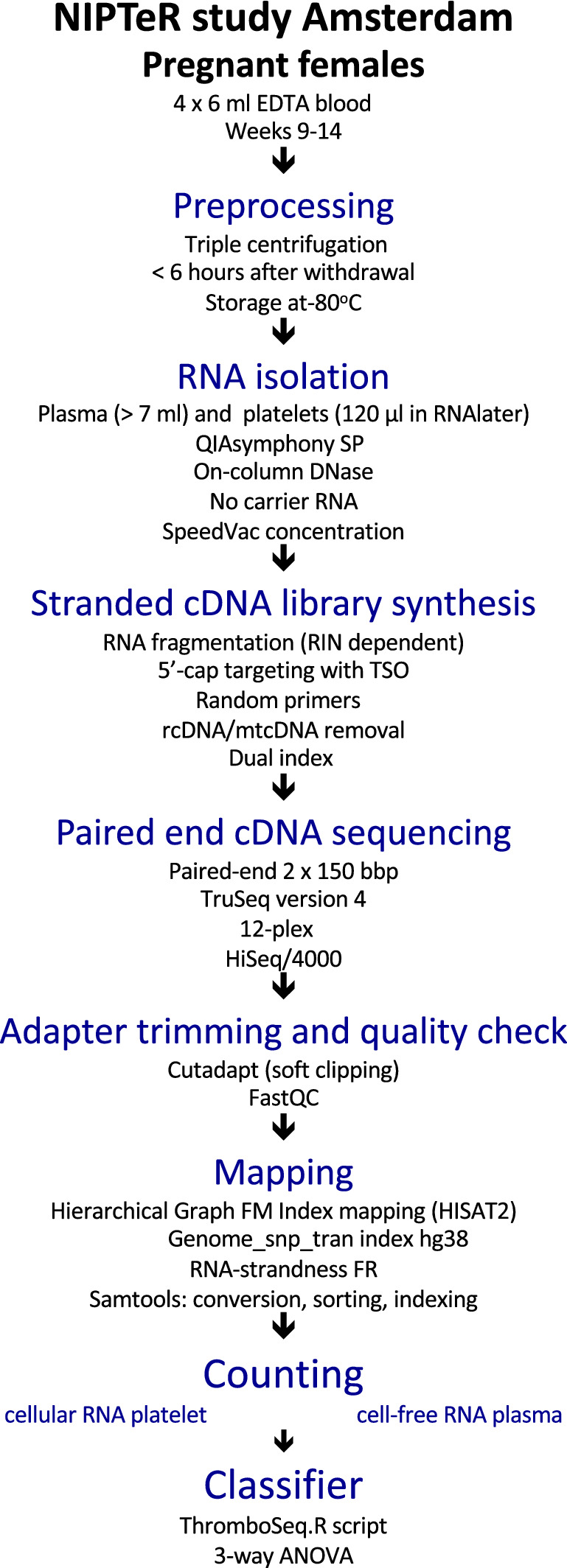
Design of the NIPTeR study. EDTA blood was collected from pregnant women during the first trimester (weeks 9–14) with informed consent and processed to obtain both cell-free fetal RNA from plasma as well as total RNA from maternal platelets from the same individuals. Total RNA was subjected to genome-wide RNA sequencing (paired end, 2 × 150 bp) of ribosomal cDNA-depleted libraries. The present manuscript describes the results of the analysis of cell-free RNA in plasma.

We obtained a first trimester (weeks 9–14) pregnancy cohort from the prospective, multicenter NIPTeR (Non-Invasive Prenatal Testing by RNA sequencing) study. The baseline characteristics and design of this study are shown in Table 1 and Fig. 1. Out of 1115 plasma samples available, a randomly chosen subset (n = 221) of this cohort was subjected to stranded, paired-end RNA sequencing of plasma RNA. This subset was subjected to RNA sequencing prior to delivery i.e. without knowledge of pregnancy outcome during selection. After collection of maternal and fetal clinical outcome following delivery of the baby, samples with twin pregnancies (including vanishing twins), trisomies and other genetic anomalies, premature rupture of membranes, loss to follow-up, withdrawn informed consent or technical failure were excluded (n = 60) (Supplementary File 1). This resulted in a cohort (n = 161) for bioinformatic analysis consisting of normal pregnancies (n = 108), affected pregnancies (n = 34) and non-pregnant controls (n = 19).

**Table 1:** Baseline characteristics of the pregnant female cohort enrolled in the NIPTeR study.

Total	1115		
**Hospital**	**# (%)**	**PHT**	**# (%)**
OLVG-Oost	122 (10.9)	No	1059 (95.0)
Academic Medical Center	386 (34.6)	Yes	41 (3.7)
VU University Medical Center	607 (54.4)	NA	15 (1.3)
**Platelets**	**# (%)**	**GA at birth**	Mean (SD)
			272.03 (18.24)
Yes	796 (71.4)		
No	26 (2.3)		
NA	293 (26.3)	**Birth weight**	Mean (SD)
			3323.46 (664.03)
**Age**	Mean (SD)	**P value**	# (%)
	33.92 (4.46)	Below_p2.3	18 (1.6)
		p3	16 (1.4)
**BMI**	Mean (SD)	p5	35 (3.1)
	24.33 (12.33)	p10	32 (2.9)
		p10–50	391 (35.1)
**GA at inclusion**	Mean (SD)	p50	79 (7.1)
	78.89 (11.14)	p50–90	417 (37.4)
		p95	68 (6.1)
**Conception**	**# (%)**	p97	12 (1.1)
Spontaneous	817 (73.3)	Above_p97	33 (3.0)
Ovulation induction	24 (2.2)	NA	14 (1.3)
IUI	60 (5.4)	**SGA**	# (%)
IVF	41 (3.7)	No	1000 (89.7)
ICSI	63 (5.7)	Yes	101 (9.1)
Cryo-embryo	80 (7.2)	NA	14 (1.3)
Oocyte donation	10 (0.9)	**Early hypertensive disorders**	**# (%)**
NA	20 (1.8)	None	1084 (97.2)
**Ethnicity**	**# (%)**	PIH	5 (0.4)
Caucasian	746 (66.9)	PIH_complaints	3 (0.3)
African	23 (2.1)	PE	10 (0.9)
Asian	29 (2.6)	HELLP	2 (0.2)
Antillian	15 (1.3)	NA	11 (10)
Moroccan	7 (0.6)	**Late hypertensive disorders**	**# (%)**
Turkish	25 (2.2)	None	1002 (89.9)
Other non-Caucasian	51 (4.6)	PIH	43 (3.9)
Surinam	18 (1.6)	PIH_complaints	28 (2.5)
Indian	17 (1.5)	PE	28 (2.5)
NA	184 (16.5)	HELLP	3 (0.3)
**Labour**	**# (%)**	NA	11 (1.0)
Induction	313 (28.1)	**SPB**	**# (%)**
PSC	167 (15.0)	< 34 week	27 (2.4)
Spontaneous	630 (56.5)	< 37 week	42 (3.8)
NA	5 (0.4)	No	1046 (93.8)
**Fetal sex**	**# (%)**		
Boy	539 (48.3)		
Girl	570 (51.1)		
NA	6 (0.5)		

NA: not-available; BMI, body mass index; GA: gestational age; IUI: intra-uterine insemination; IVF: in-vitro fertilization; ICSI: intra-cytoplasmic sperm injection; PSC: primary caesarean section; PHT: pre-existent hypertension; SGA: small-for-gestational age; PIH: pregnancy-induced hypertension; PE: pre-eclampsia; HELLP: Hemolysis, Elevated Liver enzymes, Low Platelets; SPB: spontaneous preterm birth.

Units: age: years; gestational age: days; birth weight: grams.

### Differential gene expression analysis of normal pregnancies

We started using the normal pregnancies and non-pregnant controls as reference set for differential gene expression (DGE) analysis. The normal pregnancy samples had median (interquartile range) values of maternal age of 35 years (IQR 32–37), gestational age 77 days (IQR 73–85), and uniquely assigned exonic reads 3,998,255 (IQR 2,965,559–6,023,373). Additional details of the normal pregnancies reference set are provided in Supplementary File 2 . The non-pregnant control set had median (interquartile) values of maternal age of 28 years (IQR 26–33) and uniquely assigned exonic reads 4,524,384 (IQR 3,537,585–8,5127,880).

Out of the 56,785 genes (exons: 1,237,864; exons after merge: 332,540; chromosomes: 24) analyzed as present in the gencode.v28.annotation GTF (without chrM and the RP5-857K21 cluster) and following filtering for low abundancy genes by removal of genes with less than 120 reads in more than 90% of the samples, the median numbers of genes detected and stable genes selected for RUVSeq correction were 8,565 (15.08%) and 3,717 (6.55%), respectively. Read summarization was performed at the meta-feature level (useMetaFeature = TRUE in RSubread and related packages) with all isoforms grouped together at the gene level. The genes detected represent potential biomarkers tested by DGE to identify plasma RNA markers specific for early pregnancy. The stable genes represent genes that allow correction for technical or biological confounders using RUVSeq.

The following modifications were implemented. Samples with less than 1 million uniquely-assigned exonic reads (corresponding to on average less than 100,000 fetal reads) were excluded. For this, fetal RNA fractions were determined by comparison of plasma samples from the complete set of normal pregnancies with non-pregnant controls using a total transcript approach with calculation of the RPKM ratios of placental versus reference genes. The reference genes were determined using an in-house script with the criteria set as described by Zhan et al.^7^ In first trimester normal pregnancies, the median fetal RNA fraction was 7.16% (IQR 5.11–11.04), in non-pregnant controls 0.38% (IQR 0.19–0.73). When the number of uniquely-assigned fetal reads (fetal fraction of total of unique reads) was 100,000 or less, samples were excluded (n = 8) (3.62%). This cutoff level (fetal RNA fraction < 3%) is comparable to the lower threshold of fetal DNA fractions in the NIP test^8^. Plotting of the median, lower and upper interquartile ranges (X_L_ and X_U_) of the fetal RNA fractions per gestational week showed the fetal RNA fractions to be consistent within this time window of the first trimester (Supplementary File 3).

Maternal and gestational age as well as fetal RNA fractions were subsequently implemented in the thromboSeq.R script to correct for potential confounders using RUVSeq. Potential confounding variables are corrected by selection of a panel of genes with stable expression levels among the sample cohort. RUVSeq iteratively assesses whether potential confounding factors are present in the data, and corrects for these confounding factors using the stable genes panel. The comparison of interest (pregnant versus non-pregnant) is secured from being ‘corrected’ in the dataset. The thromboSeq.R script was used with particle-swarm optimization (PSO)^9^. Details on the metaheuristic nature and other advantages of the PSO algorithm have been described elsewhere^9^. The use of PSO enhancement in our approach is limited to the optimization of the heatmap clustering by setting the threshold for FDR based on the ANOVA-analysis. Thereby, more or less RNAs are included in the heatmap improving the dendrogram’s clustering and group separation.

**Figure 2:**
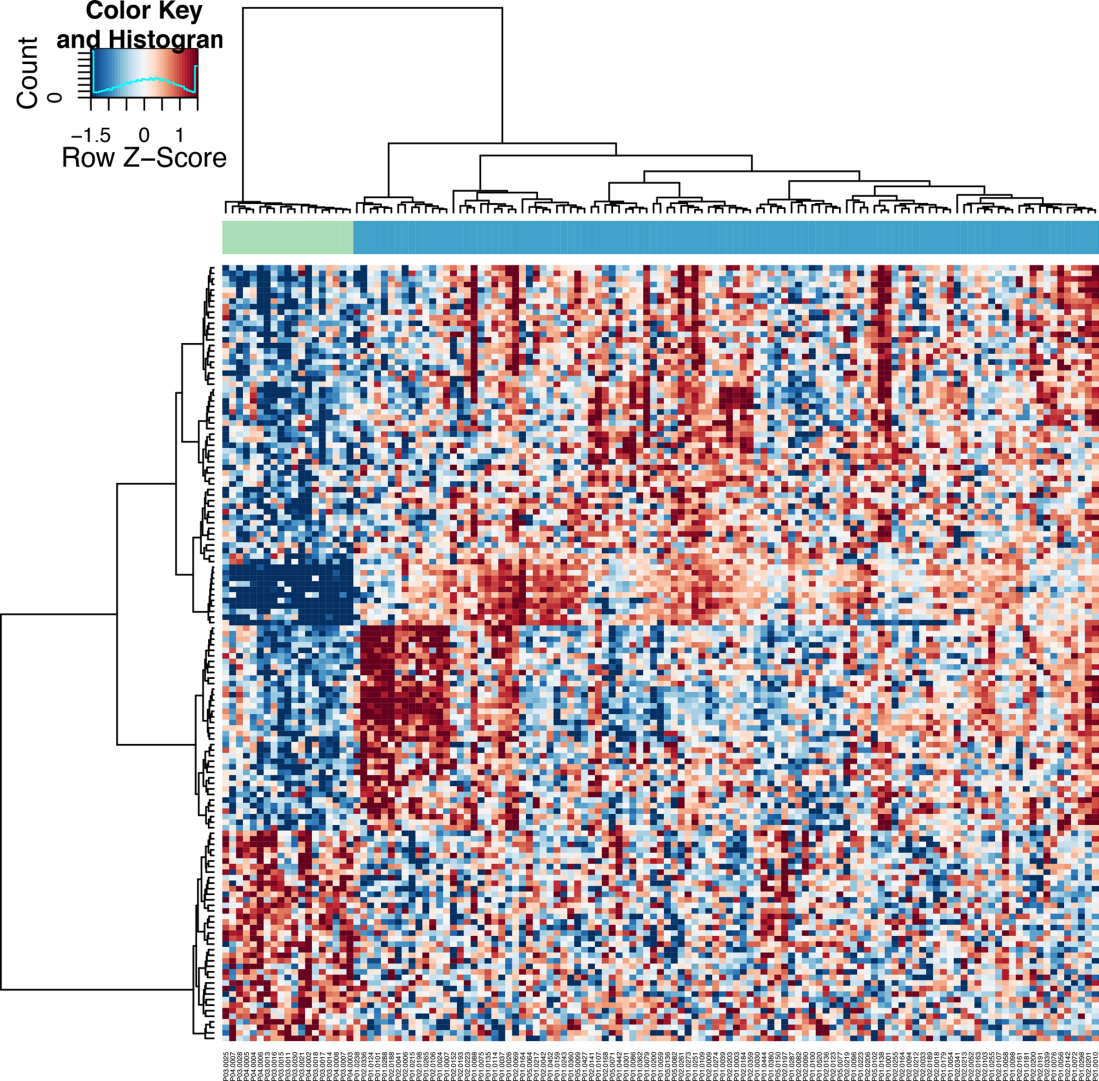
Differential gene expression analysis of normal pregnancies versus non-pregnant controls with RUVSeq correction for fetal RNA fraction and maternal and gestational age. Hierarchical clustering of differentially expressed mRNAs between pregnant (blue) and non-pregnant (green) controls. Clustering was performed with PSO-enhancement. Columns indicate samples, rows indicate genes, and color intensity represents the Z score-transformed expression values. The corresponding set of differentially expressed pregnancy-specific genes (n = 121) with significance (FDR < 0.05) are shown in Supplementary File 4.

With these optimized settings, the corresponding heatmap showed a perfect separation between pregnant and non-pregnant females (Fig. 2) with no exclusion of samples. The number of pregnancy-specific (present in pregnant samples only) or enriched (present in non-pregnant controls, but upregulated in pregnancy) genes with significance (FDR < 0.05) was 121. Eleven genes were upregulated with a logFC > 2: C*SH1, PLAC4, PSG3, CGA, PSG4, PSG1, TFPI2, VGLL3, LEP, ADAM12* and *KRT8* and 15 genes with a logFC > 1: *AC114752.3, MMP8, KCNJ2, CTB-3601.5, MS4A3, TCL6, KRT18, MMP25, CTSG, MPO, KCNJ15, TYMS, CD9, MYB, TPX2,* and *PROK2* (Supplementary File 4). Non-coding RNAs such as *LINC00649*, *LINC01272* and *MIR22HG* were correctly identified as well, but none of them were differentially expressed. MicroRNAs were identified as their larger-sized precursors with the exception of microRNAs transcribed as multicistronic transcripts such as the C19MC cluster.

Y-chromosome transcripts were below detection level in plasma at this stage of gestation (9–14 weeks) prohibiting their use as gold standard. In contrast, in pregnancies with female embryos, the *XIST* gene was significantly upregulated (logFC 2.27, FDR 0.01) compared to pregnancies with male embryos following a three group comparison (normal pregnancies with female fetuses, normal pregnancies with male fetuses, non-pregnant controls) with a 3-way ANOVA. For this observation^10^, corrections for fetal fraction and gestational age were essential, in addition to the inclusion of non-pregnant controls. The former corrects for the known interindividual- and gestational age-related variations in the levels of circulating placental nucleic acids. The latter is imperative to target the placental signal.

### VGLL3 is a pregnancy-specific marker in first trimester maternal plasma

**Figure 3:**
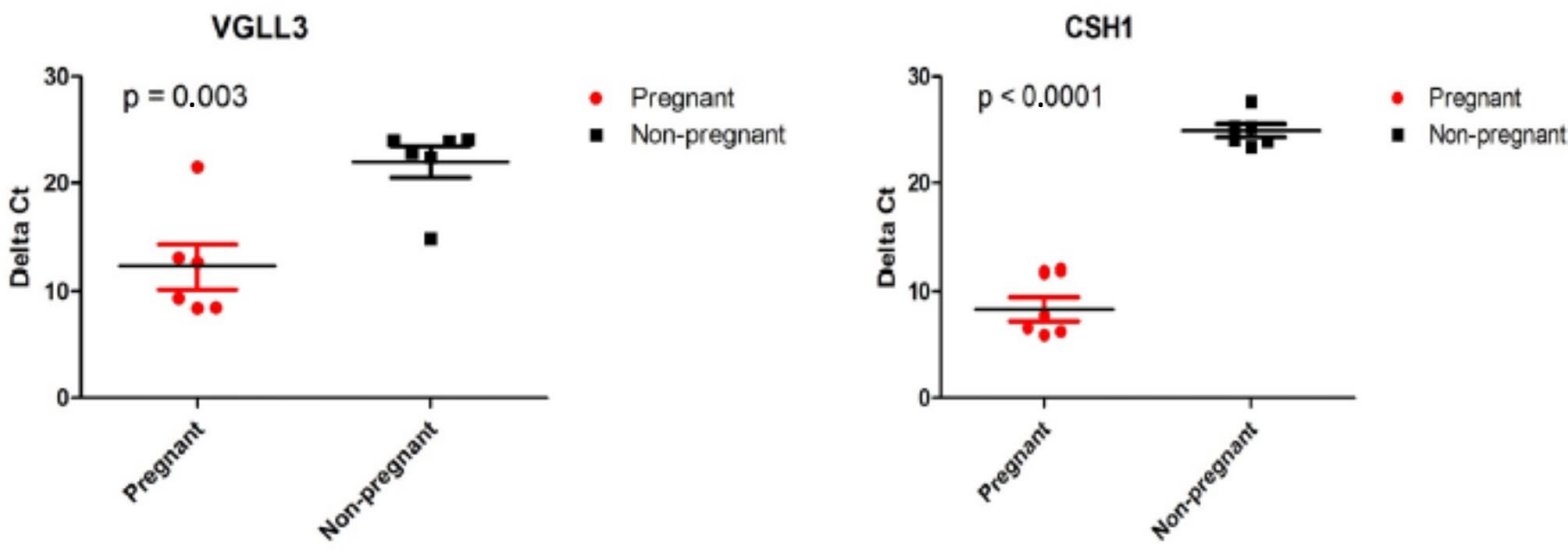
Confirmation of *VGLL3* as a pregnancy-specific marker in first trimester maternal plasma. By RT-qPCR of cell-free plasma RNA obtained from an independent first trimester cohort, *VGLL3* was confirmed to be pregnancy-specific (p-value 0.003). All pregnant samples were positive except one. *CSH1* is included for comparison.

The pregnancy-specific markers contained well known placental genes with high expression such as *CSH1* and *PLAC4*, but also included plasma markers like *VGLL3. VGLL3* is a core trophoblast gene coding for a transcription cofactor^11^. Transcription (co)factors are low in expression and nuclear in localization. These genes are inaccessible by proteomic or metabolomic assays if to be used as biomarkers in plasma for prenatal or other purposes. By RT-qPCR of cell-free plasma RNA obtained from an independent first trimester cohort, *VGLL3* was reliably detected and confirmed to be pregnancy-specific (Fig. 3). All pregnant samples were positive except one. If to be used for additional analyses, this false-negative sample, most likely caused by a low fetal RNA fraction, would have been excluded as done for RNA sequencing. Of the four members of the *VGLL* gene family, *VGLL1* is also placenta-specific, but negative in our RNA-seq analysis.

### Differential gene expression analysis of affected pregnancies

We continued by supplementing the reference sample set with samples from affected pregnancies (n = 34) (Supplementary File 5). This set contained a representative combination of prevalent pregnancy disorders: small for gestational age (SGA) (birth weight percentile < 10) (n = 10), pregnancy-induced hypertension (PIH) (n = 4), late-onset pre-eclampsia (LOPE) (n = 2), pre-existent hypertension (PHT) (n = 4), spontaneous preterm birth (SPB) (n = 13) and early-onset pre-eclampsia (EOPE) superimposed on chronic kidney failure (n = 1).

We made the following assumptions. A pathological change in placental expression is unlikely to exist in all these samples when analyzed at the presymptomatic stage. Secondly, prior to the onset of the maternal response and accompanying symptoms, the early placental pathways involved could be identical and shared between distinct clinical entities. This means that a clinical diagnosis driven approach is unlikely to be effective when analysis is done before the appearance of clinical symptoms. This can be explained by considering pregnancies with fetal growth restriction. About 70% of fetuses with a birth weight below the 10th percentile are not growth restricted, have similar uncomplicated perinatal outcomes compared to those with fetal weight in the normal range and should be considered normal variations in fetal growth (small-for-gestational age, SGA)^12^. This implies the more realistic situation in all clinical cohorts including our own, 30% of pregnancies with birth weights below the 10th percentile will be truly growth-retarded (IUGR) and have signs of (early) placental dysfunction. In other words, commonly used late clinical parameters like birth weight lack specificity and constrain informativity when used as paradigm for early pathophysiological events^12^.

To cope with this catch-22 situation, we used a metaheuristic approach for our SVM algorithm with implementation of a variant of the leave-one-out cross-validation (LOOCV) analysis. Instead of systematically leaving one sample out, we systematically added affected samples (one-by-one) to the normal reference set. With this, the primary condition of the original approach is retained: every aspect of the learning method that involves using the data—variable selection, for example—is cross-validated. In LOOCV, the observation is *removed*, the model is fit to the remaining data and this fit is used to predict the value of the *deleted* observation. In our approach, the observation (sample) is *added* and the modeled fit used to predict the value of the *added* observation. In other words, our approach can be considered the reverse of the LOOCV method. When individual samples qualified, samples were grouped to reach statistical significance. To illustrate the principle of this analysis, we have provided an explanation in Supplementary Files 6A-B and their legends.

The supervised self-learning SVM algorithm was tested accordingly. To prevent overfitting, classification, as scored by the heatmap profiles and t-tests, should comply with three criteria: (i) Pregnant versus non-pregnant discrimination should remain completely intact as visualized in the corresponding heatmaps. (ii) The group t-test should remain to show the highest significance; and (iii) The affected pregnancies should cluster as unique subgroups within the pregnancy group (discrete arms in the heatmap dendrogram).

**Figure 4:**
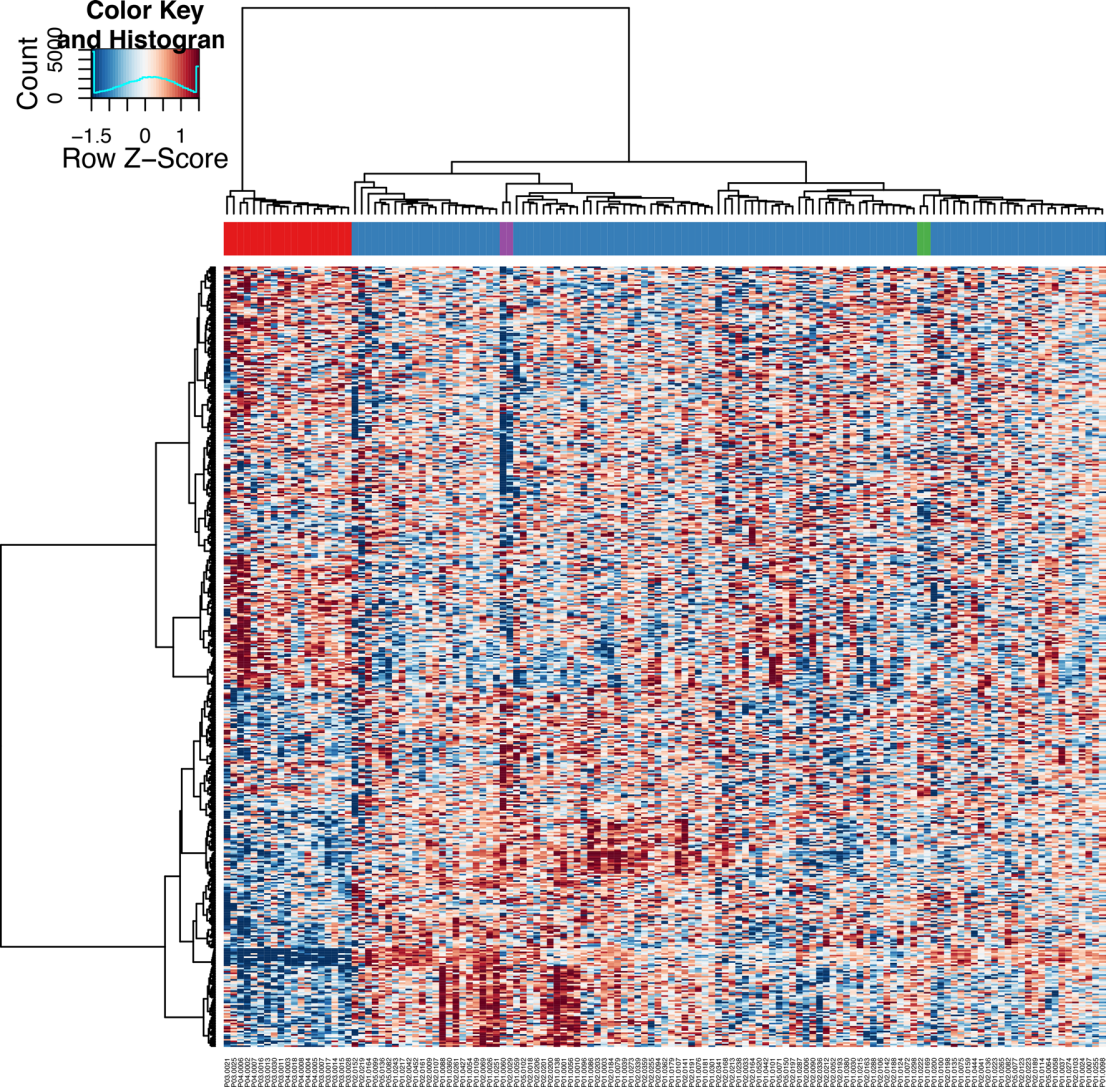
Differential gene expression analysis of affected pregnancies. Hierachical clustering of differentially expressed mRNAs between normal pregnancies,(blue), non-pregnant controls (red) and affected pregnancies with fetal (green) or maternal (purple) origin (Supplementary File 5). Clustering was performed with PSO-enhancement. Columns indicate samples, rows indicate genes, and color intensity represents the Z score-transformed expression values.

With this approach, when we performed a 4-group comparison with a 4-way ANOVA, four samples were identified, two in each subgroup (Fig. 4) where all three criteria were met. One subgroup involved two affected pregnancies with presumably primary fetal origin (P01-0169, P01-0222): both had fetal growth restriction (birth weight percentile p5) as sole complication. In the second subgroup with presumably primary maternal origin, one affected pregnancy (P01-0060) had pregnancy-induced hypertension complicated by IUGR (< p2.3) and premature delivery at week 36. The other pregnancy with maternal origin (P02-0029) had pre-existent hypertension with premature delivery at week 28.

### Identification of NRIP1 as a potential biomarker for pre-eclampsia

Using the same add-one-in approach as above, we identified a unique pattern for the sample with early-onset pre-eclampsia superimposed on chronic kidney failure (P01-0278). A highly aberrant gene profile was seen with segregation as a distinct subgroup yet in combination with a single *non-pregnant* control sample (P03-0021) (Supplementary File 7). This non-pregnant sample had no clinical or family history, or clinical evidence for an underlying disease (such as renal failure, medicine use), and was confirmed not to be pregnant when the blood sample was obtained. When the sequence files of these 2 samples were analyzed with Taxonomer, a metagenomics on-line software package for microorganism detection in NGS data (www.taxonomer.com), bacterial sequences (species: Pseudomonas) (> 7.5%) were identified in both. Despite this interference with a non-specific signal unrelated to pregnancy, the ANOVA table of the aberrant gene profile remained pregnancy-specific. This included significant upregulation of *NRIP1* (logFC 1.433147, p-value 0.0004529, FDR 0.00744719) and significant downregulation of *ZEB2* (logFC -0.65829797, p-value 0.00053171, FDR 0.00053171).

The samples with spontaneous preterm birth (SPB) as sole clinical complication were non-informative and all segregated with the normal pregnancy samples, both alone and in combination and irrespective of the cutoff taken (< 37 weeks or < 34 weeks). When analysis was limited to the set of 51 transcripts reported to be informative in late pregnancy^4^, the results remained non-informative in the comparison between normal and SPB pregnancies. This suggests that, although SPB can be classified up to two months in advance of labor, presymptomatic classification appears to be restricted to late second or early third trimester^4^.

When the SPB cases are excluded, 5 out of 21 affected pregnancies (23.8%) showed aberrant transcriptome profiles in maternal plasma at the presymptomatic stage. Of the pregnancies with fetal birth weights below the 10th percentile (n = 11), three (27.3%) had distinct presymptomatic profiles. This percentage would correlate with the percentage of true cases with intrauterine growth retardation as predicted by the nationwide, multicentre, stepped wedge cluster randomised trial in the Netherlands^12^.

### Validation of RNA biomarkers for affected pregnancies assessment

#### Quantitative analysis of AVPR1A, PRKG1 and ARPIN

The ANOVA table of the 4-group comparison contained potential presymptomatic RNA biomarkers suggestive for a dysfunctional pathway operating early in the disease process of a subset of clinically distinct pregnancy disorders with growth restriction as a shared symptom. Out of the genes with significance (− 2 < logFC > 2 and p < 0.001), we arbitrarily selected three genes (*PRKG1, AVPR1A*, *ARPIN*) for validation by RT-qPCR in plasma samples from an independent first trimester cohort (Supplementary File 8). *AVPR1A* and *PRKG1* correlate with regulation of vascular smooth muscle cell contraction (KEGG hsa04270)^13^. *ARPIN* is associated with negative regulation of cell migration. Both processes are important in the placental bed in the first trimester and dysregulated in affected pregnancies. Both *PRKG1* and *AVPR1A* showed a dichotomous pattern with two discrete groups with normal versus downregulated expression profiles, while *ARPIN* did not. *AVPR1A* showed the presence of false-positives (reduced signal in normal uncomplicated pregnancies), while *PRKG1* had no false-positives and was statistically significant (p-value 0.0356) (Supplementary File 9).

#### Quantitative analysis of *NRIP1* and *ZEB2*

As shown above, the *NRIP1* and *ZEB2* genes were differentially expressed in the pregnant samples with early-onset pre-eclampsia. *NRIP1* has been previously identified as the only gene consistently upregulated in the analysis of all RNA-seq studies (microarray, RNA-seq, PAS-seq) of pre-eclamptic placentas^14^. Secondly, *ZEB2* is functionally directly and inversely related with *NRIP1*. *ZEB2* mediates trophoblast differentiation (villous to extravillous trophoblast differentiation) by regulating epithelial-mesenchymal transition and is similar to *NRIP1,* a key transcription factor in pre-eclampsia with differential expression (downregulation) in affected placentas^14–17^.

However, the placental upregulation of *NRIP*1 in pre-eclamptic placentas as analyzed at the symptomatic stage is inconsistent with a role in the etiology of pre-eclampsia. *NRIP1* is located on chromosome 21 and upregulated in trisomy 21, including upregulation in the placenta^18^. Trisomy 21 pregnancies, however, do not show an increased risk for pre-eclampsia; the crude or confounder-adjusted OR for having preeclampsia in relation to trisomy 21 is 0.63 (95% CI 0.47–0.85)^19^.

**Figure 5:**
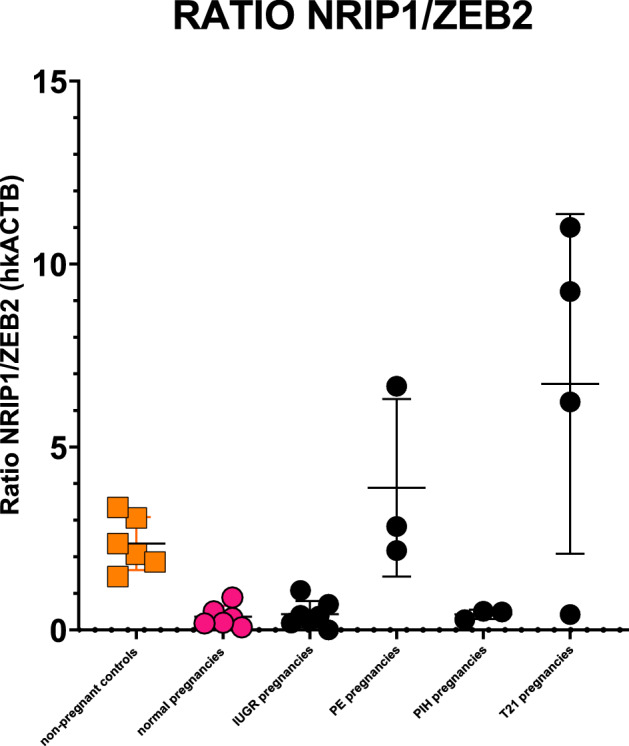
First trimester NRIP1/ZEB2 plasma ratio's are significantly upregulated in pregnancies complicated by pre-eclampsia and in pregnancies with trisomy 21 fetuses. Normalization was done using *ACTB* (actin B). IUGR: intrauterine growth retardation; PE: pre-eclampsia; PIH: pregnancy-induced hypertension; T21: trisomy 21 (karyotypically confirmed).

We quantified *NRIP1* and *ZEB2* by RT-qPCR in the same independent sample set as above and supplemented this set with first trimester plasma samples from karyotypically confirmed trisomy 21 cases. In pregnancies complicated by pre-eclampsia or trisomy 21, *NRIP1* levels when analyzed either alone or as *NRIP1/ZEB2* ratio, increased significantly compared to normal uncomplicated pregnancies (Fig. 5, Supplementary File 10).

This overlap would prevent reliable discrimination between pre-eclampsia and trisomy 21 pregnancies if to be used for clinical diagnostics. However, following Sanger sequencing of the PCR amplicons from the samples with pre-eclampsia and trisomy 21, we noticed the presence of circular *NRIP1* in addition to linear *NRIP1* in these samples. Apparently, the use of random primers during first strand synthesis generates both RNA forms, both linear and circular RNA. In addition, we recently demonstrated the presence and importance of circular *NRIP*1 as a pregnancy-specific RNA biomarker^20^.

We therefore performed quantification for the circular forms of *NRIP1* as well to discriminate between the relative quantities of linear and circular *NRIP1* (ratio's) in the samples with pre-eclampsia and trisomy 21. This approach was found to permit discrimation between cases of pre-eclampsia and trisomy 21. Pre-eclamptic pregnancies showed an increase in linear *NRIP1* compared to circular *NRIP1*, while T21 pregnancies did not (Supplementary File 11).

## Conclusion

Here we show that, by using a metaheuristic approach, RNA biomarkers can be identified with the potential to be used as presymptomatic markers for affected pregnancies. The outcome of our study is unexpected when considered from a conventional clinical point of view for the following reason. We show that analysis of affected pregnancies at an early stage in the disease process, i.e. prior to the appearance of clinical symptoms, is more effective when analysis is focused on similar symptoms (e.g. growth restriction) shared between different clinical entities (i.e. pre-eclampsia, PIH, growth restriction) rather than focusing on the combination of symptoms (e.g. hypertension, proteinuria, edema) unique for distinct clinical entities (i.e. pre-eclampsia). This can be explained by the multifactorial and multisequential nature of most pregnancy-associated diseases. Prior to the onset of the late maternal pathophysiological phenomena, and their accompanying secondary pathways involved, the early (placental) pathways could be shared or identical. The finding of shared pathways, operating at an early stage in a disease process, is rather becoming a rule instead of an exception, as supported by the evidence from genome-wide screening assays. For example, in a recent comprehensive cancer study involving comparative whole-genome DNA sequencing of tumor and normal tissue, novel and unexpected pathways were found to be identical between different clinical entities^21^.

In summary, based on the results of our proof-of-principle study we propose that a metaheuristic approach as outlined in the present study is to be preferred over a clinical diagnosis-driven approach for the identification of the early pathophysiological pathways shared between prevalent pregnancy-associated disorders. Secondly, the bivariate *NRIP1/ZEB2* RNA marker is informative for presymptomatic screening of pre-eclampsia provided that the relative quantities of both the linear and circular forms of *NRIP1* are analyzed using quantitative assays that discrimate between both forms. Future large-scale screening studies should therefore not only include larger samples numbers, but also should include the extended approach with inclusion of circNRIP1 analysis. This will enable independent research groups to explore the validity of our claim.

Finally, *VGLL3* can be included as a sensitive placenta marker to rule out false-negativity as a consequence of low fetal RNA fractions.

## Materials and methods

### Study population

The NIPTeR (Non-invasive Prenatal Testing by RNA sequencing) study is a prospective, multicenter study (NL48929.029.1). Participants were women attending the antenatal units of one of three hospitals (VU University Medical Center, Academic Medical Center and OLVG Oost) in the greater Amsterdam region in the Netherlands. Inclusion criteria were an intact intra-uterine pregnancy, confirmed by ultrasound within the last 2 weeks before the blood sample was drawn, and gestational age between 9^+0^ and 13^+6^ weeks. Exclusion criteria were inability to read Dutch or English, age < 18 years and having had invasive diagnostics (chorionic villus biopsy) within the last 2 weeks. After informed consent blood samples (4 × 6 ml EDTA) were taken. Pregnancies generated by assisted-reproduction (IVF, ICSI, IUI) were included. Ethical approval was obtained from local (Medical Ethical Committees VU University Medical Center and Academic Medical Center, Amsterdam) and national (CCMO-ABR) ethical committees. Monitoring was done by the Clinical Research Bureau on a regular basis, and data storage was done in Open Clinica with a strict separation between study number and patient identification and information. All methods were performed in accordance with the relevant guidelines and regulations. Personnel involved was qualified according to GLP standards and the laboratories involved are ISO-norm (ISO-15189) accredited, both for diagnostics and research.

### Sample processing

EDTA blood (4 × 6 ml) was stored at 4 °C and processed within 6 h after withdrawal by using an adapted triple-centrifugation step to obtain plasma and platelet fractions from the same blood sample^22^. After centrifugation for 20 min at 120 × g and 4 °C, platelet rich plasma was collected and subjected to a second centrifugation step for 20 min at 360 × g and 4 °C. The supernatant plasma was carefully removed without disturbing the platelet pellet. The platelet pellet was resuspended in 120 µl RNAlater (AM7020, Thermo Fisher Scientific) and following overnight storage at 4° C stored at − 80 °C. The plasma supernatant was subjected to the third centrifugation step for 25.000 × g at 4 °C. The plasma supernatant was collected and stored at − 80° C.

### RNA isolation

RNA was isolated from plasma (> 7 ml/sample) using the QIAsymphony SP robot (Qiagen) from randomly selected plasma samples according to the manufacturer's instructions except that an on-column DNase step was included. Eluted RNA was concentrated by Savant SpeedVac SPD111V Concentrator (Thermo Scientific).

### cDNA library construction

cDNA libraries were generated using the SMARTer stranded total RNA-Seq kit (pico input mammalian) (Cat No. 635007) according to the manufacturer's instructions with the exception of replacing the reverse primer with an N6-oligo primer lacking the oligo-dT primer. RNA denaturation time for plasma samples was 4 min for all samples. Final PCR amplification was done for 16 cycles. Ribosomal cDNA was removed using ZapR treatment with R-probes. Purification was done with Agencourt AMPure XP beads (A63881, 60 ml) (Beckman Coulter). Following Agilent Bioanalyzer profiling (Agilent RNA 6000 Pico kit) (Cat. No 5067-1513), cDNA libraries were pooled in equimolar amounts.

### RNA sequencing

High throughput sequencing (paired end) was done on the HiSeq2500 (2 × 125 bp) or HiSeq4000 (2 × 150 bp) using TruSeq chemistry (version 4) with 12 samples (dual index) per lane (12 plex). PhiX was spiked at 1 or 5%.

### Data processing

Following demultiplexing (bcl2fastq), paired FASTQ files were adapter-trimmed with soft clipping (Cutadapt-v1.9), quality checked (fastQC-v.0.11.5 or fastP) and mapped (Hisat-v2.1.0) with an in-house generated hg38 genome index including SNP and transcript information. Sorted (both name and chromosome) and indexed .bam files (Samtools-v1.8) were checked for consistency and quality using IGV-v2.5.0 and QoRTs (v1.3.6).

### Sample selection

After sequencing and determination of pregnancy outcome, samples with twin pregnancies (including vanishing twins), trisomies and other genetic anomalies, premature rupture of membranes, other anomalies, loss to follow-up or technical failure were excluded from bioinformatic analysis.

### Read counting

Counts of uniquely assigned fragments were calculated with FeatureCounts (RSubread) as implemented in Verse (https://github.com/qinzhu/VERSE) using the gencode.v28.annotation GTF without chrM and the RP5-857K21 cluster. Analysis was done at the gene level (meta-feature). Additional settings included: feature type: exon, paired-end: yes, strand-specific: yes, multimapping reads: not counted, chimeric reads: counted, both ends mapped: not required.

### Differential gene and transcript expression analysis

The read count output was fed to a modified version of the thromboSeq.R script using RStudio (1.1.423) (https://github.com/MyronBest/thromboSeq_source_code)9. We included confounder-dependent correction (RUVseq) for multiple variables known to have an impact on the outcome of pregnancy samples such as gestational age and fetal RNA fraction. Fetal RNA fractions were determined as described below. The total number of corrections implemented was 5 (maternal age, library size, gestational age, fetal RNA fraction, purification). The threshold for cross-correlation was set at 0.5. Other settings were: selectBiomarkers FDR = TRUE, removal of genes with less than 120 reads in more than 90% of samples, and threshold for median number of genes = 750.

### Fetal RNA fraction determination

Fetal RNA fractions were determined by comparison of plasma samples from normal pregnancies with non-pregnant controls using a total transcript approach: RPKM ratios of placental genes versus reference genes. The placental genes (FDR < 0.05) were determined using thromboSeq.R^9^. The reference genes were determined using an in-house script with the criteria set as described by Zhan et al.^6^ Genes with RPKM values with a mean of < 1 in all samples, a mean difference of < 0.8 or > 1.2 after comparison of pregnant versus non-pregnant controls and average CV values of more than 0.3 were excluded as reference genes. Fetal fractions were subsequently determined by (mean RPKM placental genes/mean RPKM reference genes) × 100.

### RT-qPCR analysis

Candidate biomarker genes were validated by RT-qPCR using plasma samples from an independent cohort of first trimester maternal plasma samples collected in our hospital between 2003 and 2012^22^. RNA was isolated from 4 ml on the QIAsymphony SP using the QIAsymphony DSP Virus/Pathogen Kit with on-column DNase treatment. Elution was in nuclease-free water (50 µl). Using 10 µl of eluted RNA (corresponding to 800 µl plasma) as input, cDNA was generated using the Maxima H Minus First Strand cDNA Synthesis Kit (Thermo Fisher Scientific, K1652) with random hexamer primers. The cDNA was subsequently preamplified by combining TaqMan PreAmp Master Mix (2x) (Applied Biosystems, 4,384,267), pooled assay mix (combined Taqman assays to a final concentration of 0.05X per assay) and 10 µl of cDNA. Preamplication was done with 10 min of enzyme activation (95 °C), 14 cycles of 15 s denaturation (95 °C) and 4 min of annealing (60 °C) followed by 10 min of enzyme inactivation. qPCR was done using TaqMan Gene Expression Assays (Applied Biosystems) , preamplified cDNA products (diluted 1:20), master mix (2x) (TaqPath qPCR master mix, CG, A15297) and nuclease-free water in a total volume of 20 µl. The qPCR was performed by 2 min of UNG incubation (50 °C), 20 secs of enzyme activation (95 °C) and 40 cycles of 1 s denaturation (95 °C) and 20 s of annealing (60 °C). Each gene was analyzed in duplicate. Analysis was done using the Rotor-Gene Q software and delta Ct analysis. *ACTB* or *TOP1* were used as reference genes. Details on the TaqMan gene expression assays used are available on request.

Following Sanger sequencing of the PCR amplicons of the samples from pregnancies with pre-eclampsia or trisomy 21 fetuses, we noticed the (variable) presence of circular *NRIP1* in addition to linear *NRIP1*. We therefore complemented the quantitative analysis of linear NRIP1 with quantitative analysis of circular *NRIP1* in these samples using the preamplified cDNA products. Details on the analysis of circ*NRIP1* have been described elsewhere^20^.

## Supplementary information

Supplementary Information 1.

## Data Availability

All sequencing data are available on NCBI SRA, accession SUB7550002.
