# Supplementary material for: The bivariate NRIP1/ZEB2 RNA marker permits non-invasive presymptomatic screening of pre-eclampsia
Source: Sci Rep. 2020 Dec 14;10:21857. doi: 10.1038/s41598-020-79008-4 (PMC7736279; doi:10.1038/s41598-020-79008-4)
Supplement: Supplementary file 1 — Supplementary Information 1. [file 41598_2020_79008_MOESM1_ESM.pdf]

SUPPLEMENTARY INFORMATION

THE BIVARIATE NRIP1/ZEB2 RNA MARKER PERMITS NON-INVASIVE PRESYMPTOMATIC SCREENING OF PRE-ECLAMPSIA

Vera Manders[1,2], Allerdien Visser[1], Remco Keijser[2], Naomi Min[1,2], Ankie Poutsma[1], Joyce Mulders[1], Tarah van den Berkmortel[1], Marjolein Hortensius[1], Aldo Jongejan[3], Eva Pajkrt[4], Erik A Sistermans[5], Daoud Sie[5], Myron G Best[6,7,8], Tom Würdinger[6,8], Marjon de Boer[9], Gijs Afink[2], Cees Oudejans[1*]

[1]Department of Clinical Chemistry, Amsterdam UMC, VU University Medical Center, Amsterdam, The Netherlands
[2]Reproductive Biology Laboratory, Amsterdam UMC, Academic Medical Center, Amsterdam, The Netherlands
[3]Department of Clinical Epidemiology, Biostatistics and Bioinformatics, Amsterdam UMC, Academic Medical Center, The Netherlands
[4]Department of Obstetrics/Gynecology, Amsterdam UMC, Academic Medical Center, Amsterdam, The Netherlands
[5]Department of Clinical Genetics, Amsterdam UMC, VU University Medical Center, Amsterdam, The Netherlands
[6]Department of Neurosurgery, Amsterdam UMC, VU University Medical Center, Amsterdam, The Netherlands
[7]Department of Pathology, Amsterdam UMC, VU University Medical Center, Amsterdam, The Netherlands
[8]Brain Tumor Center Amsterdam, Amsterdam UMC, VU University Medical Center, Amsterdam, The Netherlands
[9]Department of Obstetrics/Gynecology, Amsterdam UMC, VU University Medical Center, Amsterdam, The Netherlands

Supplementary file 1: First trimester plasma samples used for differential gene expression analysis. Following exclusion for technical or clinical reasons, 161 maternal plasma RNA samples of normal pregnancies, affected pregnancies and non-pregnant controls qualified for differential gene expression analysis.

NIPTeR STUDY: MATERNAL PLASMA RNA SEQUENCING

**A**	Total number of plasma samples processed for RNA-seq			221
**B**	Clinical exclusions	Retracted informed consent		24
		No follow up available		15
		Twin pregnancy, PROM, genetic anomaly		8
**C**	Technical exclusions	Low fetal fraction		8
		Low counts		5
**D**	Samples used for DGE	**Normal**		108
		**Affected**	Spontaneous preterm birth	13
			Early-onset pre-eclampsia	1
			Intra-uterine growth restriction	10
			Late-onset pre-eclampsia	2
			Pre-existent hypertension	4
			Pregnancy-induced hypertension	4
		**Non-pregnant controls**		19
**E**	Total number of samples processed for DGE analysis			**161 (72.9 %)**

Supplementary File 2. Additional details of the normal pregnancy reference set.

Additional details of the normal pregnancy reference set (n=108)

Average gravidity		2
Average parity		1
Conception	Normal	66
	Assisted reproduction	42
Etnicity female	Caucasian	88
	Non-caucasian	20
Consanguinity		None
Average height		170 cm
Average weight		68 kg
Average BMI		23.5
Smoking		None
Alcohol		None
Drugs		None
Early hypertensive disorders		None
Late hypertensive disorders		None
Small-for-gestational age		None
Pre-existent hypertension		None
Spontaneous preterm birth		None
Twin pregnancies		None
Miscarriage		None
Termination of pregnancy		None
Preterm delivery		None
Median (IQR) gestational age at birth		278 (272-283) days

Supplementary file 3: Consistent levels of fetal RNA fractions in weeks 9-14 of pregnancy. Fetal RNA fractions were determined by comparison of plasma samples from normal pregnancies with non-pregnant controls using a total transcript approach: RPKM ratios of placental genes versus reference genes. The reference genes were determined using an in-house script with the criteria set as described by Zhan *et al.*[6] Median and lower and upper interquartile ranges (X_L and X_u) of the fetal RNA fractions per gestational week are indicated.

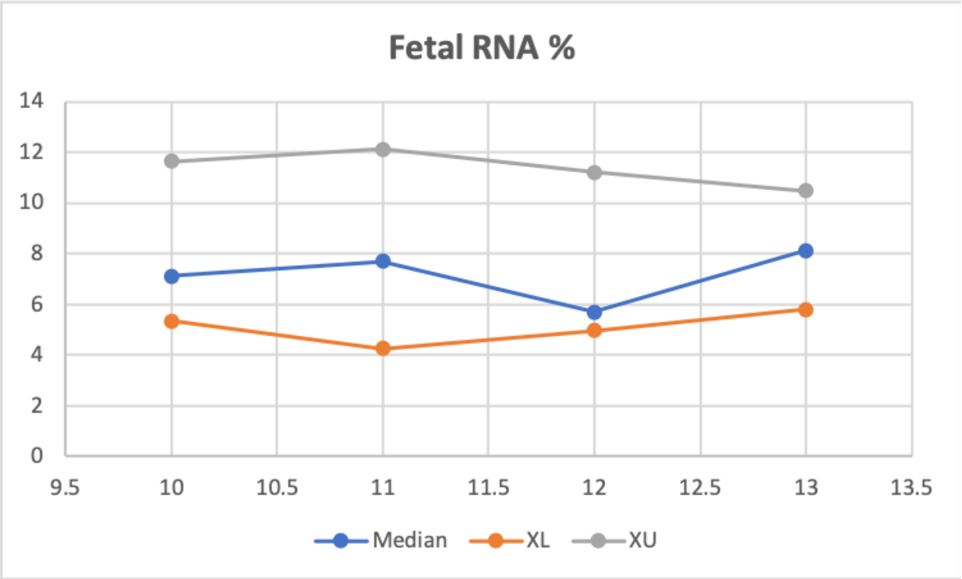
Fetal RNA %
14
12
10
8
6
4
2
0
9.5
10
10.5
11
11.5
12
12.5
13
13.5
Median
XL
XU

Supplementary file 4: Pregnancy-specific genes with differential expression in first trimester maternal plasma compared to non-pregnant controls. Abbreviations: logFC, logarithm of FoldChange; logCPM, logarithm of counts per million; LR, likelihood ratio; pValue, probability value; FDR, False Detection Rate.

genes	logFC	logCPM	LR	PValue	FDR
CSH1	7.15963967	4.94642832	226.049202	4.33E-51	3.71E-47
PLAC4	8.21746526	8.35162049	177.545426	1.66E-40	7.13E-37
PSG3	7.16540483	3.92036345	113.498633	1.68E-26	4.79E-23
CGA	5.51843328	5.75101501	94.7443708	2.17E-22	4.64E-19
PSG4	5.53698572	4.39946592	80.8357694	2.45E-19	4.20E-16
PSG1	5.93145791	4.36903282	76.5662327	2.13E-18	3.04E-15
TFPI2	3.7755101	5.11599161	69.0674967	9.52E-17	1.16E-13
VGLL3	3.60483423	4.70923025	63.970408	1.26E-15	1.35E-12
LEP	4.97558443	3.7023532	50.2579239	1.35E-12	1.28E-09
ADAM12	2.79152486	3.85131644	49.1196871	2.41E-12	2.06E-09
KRT8	2.28582594	4.49139351	42.0372579	8.96E-11	6.97E-08
NAMPT	0.82207567	9.60960108	32.4944672	1.20E-08	8.53E-06
ACSL1	0.89675692	7.14221149	29.2857009	6.25E-08	4.11E-05
SOD2	0.62077488	8.78285656	28.5264031	9.24E-08	5.65E-05
AC018890.1	0.94475831	5.78774123	28.0514874	1.18E-07	6.75E-05
SERF2	0.49217203	7.60319475	27.4516149	1.61E-07	8.62E-05
CREB5	0.81300565	6.16584145	26.510778	2.62E-07	0.00013204
SRGN	0.78926615	7.36699507	25.71643	3.95E-07	0.00018817
S100A9	0.98128801	10.2257516	25.6031162	4.19E-07	0.00018904
AC002480.6	-0.6875699	5.63465967	25.4122003	4.63E-07	0.00019827
CXCR2	0.90561613	6.19901171	24.9242952	5.96E-07	0.00024319
PFKFB3	0.68825114	5.57444159	23.570949	1.20E-06	0.00046871
TPX2	1.13321319	4.71129375	22.7294067	1.86E-06	0.00069448
PDCD4	-0.3102213	8.7388604	22.0066639	2.72E-06	0.00096965
FAM129A	0.55350079	7.43976037	21.7692273	3.07E-06	0.00105347
FKBP1A	0.45835925	6.87887178	21.4614946	3.61E-06	0.00118924
CIT	0.97429743	6.19543155	21.1497181	4.25E-06	0.00134744
S100A8	0.98476243	8.90143018	20.7692709	5.18E-06	0.00158476
LIMK2	0.76571016	5.28762448	20.400767	6.28E-06	0.0018549
KRT18	1.34481258	4.04781931	20.3085785	6.59E-06	0.00188157
ZNF326	-0.3959194	6.91232261	19.9519763	7.94E-06	0.00219407
IFITM2	0.46101298	7.7104502	19.6117734	9.49E-06	0.0025396
MMP25	1.2521393	3.52740753	19.4223701	1.05E-05	0.00271933
SERPINB1	0.46066436	8.04999796	19.1776936	1.19E-05	0.00291938
TYMS	1.20414274	4.48077735	19.1744801	1.19E-05	0.00291938
L29074.1	0.73852371	5.14043176	18.6071464	1.61E-05	0.00382135
MS4A3	1.47096576	5.23464508	18.5125224	1.69E-05	0.0039073
ANKRD13D	0.64625267	5.35792303	18.3174357	1.87E-05	0.00410381
SUMO3	0.37247546	6.56280091	18.2339534	1.95E-05	0.00410381
KCNJ2	1.61013656	3.58593784	18.18983	2.00E-05	0.00410381
ETS1	-0.2596313	9.75030994	18.178446	2.01E-05	0.00410381
MYB	1.16671861	5.16807624	18.1712857	2.02E-05	0.00410381
ANXA3	0.87967424	6.74309474	18.1327366	2.06E-05	0.00410381
MMP8	1.76739775	5.41900634	17.9758153	2.24E-05	0.0043551
AQP9	0.93504509	5.54999738	17.9201038	2.30E-05	0.0043848

BASP1	0.55971425	7.05655146	17.5448648	2.81E-05	0.00522479
TBCK	-0.7272044	4.68894944	17.4686617	2.92E-05	0.00532274
TCL6	1.44635788	3.88396674	17.3919276	3.04E-05	0.00542655
GCA	0.57402114	7.80053868	17.2314013	3.31E-05	0.00578437
FYB1	0.29512409	10.5611359	16.9770579	3.78E-05	0.006481
ARF1	0.30888369	8.14309189	16.7578881	4.25E-05	0.00713168
KCNJ15	1.21499173	4.41182233	16.6079715	4.60E-05	0.00748927
CD37	-0.3439749	8.17550473	16.5921093	4.63E-05	0.00748927
H3F3A	0.46286411	9.06144137	16.4929635	4.88E-05	0.00774513
POU2AF1	-0.6023881	5.80677509	16.2452016	5.57E-05	0.00866238
MPO	1.24271084	4.73999591	16.1824252	5.75E-05	0.00866238
IGHM	-0.4697644	7.41622183	16.1783879	5.76E-05	0.00866238
CDK2AP1	0.77657455	5.28504035	16.0178671	6.27E-05	0.00926608
GPD2	0.79557098	5.58297091	15.9831157	6.39E-05	0.00927777
TNFAIP2	0.41866804	7.09152142	15.7947885	7.06E-05	0.01007768
NCF2	0.43529748	8.70556399	15.634535	7.68E-05	0.01078886
PGD	0.38166329	7.97177667	15.589003	7.87E-05	0.01087355
H3F3B	0.2459624	8.47937079	15.3947252	8.72E-05	0.01185932
NRBF2	0.45300638	6.47521151	15.1034626	0.00010178	0.01361356
LITAF	0.33902014	8.17080874	15.0751639	0.00010331	0.01361356
TPM1	0.69953553	7.19401759	15.0337391	0.00010561	0.01370481
TECPR2	0.50550058	5.91338857	14.8902287	0.00011395	0.014419
STX3	0.62449647	5.92220972	14.8815591	0.00011448	0.014419
CSF2RB	0.82586689	4.86442618	14.8064325	0.00011913	0.01478747
CD44	-0.2741135	8.47533785	14.6501674	0.00012942	0.01583585
TET2	0.31149033	8.5054508	14.6030094	0.0001327	0.0160084
RAB31	0.52245292	7.96647523	14.3946799	0.00014822	0.01763206
MSRB1	0.72706213	5.38043752	14.2608924	0.00015914	0.01867137
TCF25	-0.4583644	5.72902864	13.9228791	0.00019047	0.02175696
DYSF	0.48023668	7.29152609	13.9172773	0.00019103	0.02175696
NELFE	0.54259839	5.26728858	13.8974862	0.00019306	0.02175696
BIRC2	-0.5026184	8.17651854	13.6407841	0.00022132	0.02446872
MXD1	0.51057837	7.70900046	13.6053299	0.00022554	0.02446872
PRRC1	-0.5130367	5.34860755	13.5773026	0.00022894	0.02446872
MYL6	0.33123003	9.17015476	13.5565368	0.00023148	0.02446872
PROK2	1.02180388	4.61793638	13.5498689	0.00023231	0.02446872
NFE2	0.45880343	6.30023219	13.5341634	0.00023426	0.02446872
CDA	0.7388446	4.770739	13.4347834	0.000247	0.02548877
RCAN3	-0.3623353	7.03889604	13.389395	0.00025305	0.02580216
PLEK	0.31946559	8.90131968	13.3448876	0.00025913	0.0260572
ZNF185	0.86434459	6.62999228	13.3268201	0.00026164	0.0260572
HMGB3	0.8261885	4.49933831	13.2973415	0.00026578	0.02616585
ZNF445	-0.3988931	6.13729641	13.2747245	0.00026901	0.02618247
PRKCSH	-0.3033269	7.06687829	13.2291819	0.00027562	0.0262982
MNDA	0.40828659	10.0766191	13.2243311	0.00027634	0.0262982
POLR3D	-0.4728752	5.35926075	13.1772159	0.00028337	0.02667136

LMNB1	0.4055708	6.90142389	13.0517492	0.000303	0.02820255
CTB-36O1.5	1.58782593	3.41356783	13.0319143	0.00030623	0.02820255
GAPDH	0.24142829	9.55794109	12.7181137	0.00036213	0.03299628
RAD54L2	-0.3321939	6.34400913	12.6426413	0.00037705	0.03399375
RNASEH2C	0.84102001	4.06115608	12.5827047	0.00038933	0.03455569
MYC	-0.4071716	5.86813314	12.5419988	0.00039791	0.03455569
MAX	0.54107041	8.62696127	12.5419966	0.00039791	0.03455569
SNX29	-0.357832	6.65434973	12.534915	0.00039942	0.03455569
SLMAP	0.39741206	7.42110923	12.5093664	0.00040492	0.03468113
H2AFX	0.82473329	4.22541277	12.4509004	0.00041779	0.03537569
PRKCD	0.38774596	6.97347882	12.4353351	0.00042129	0.03537569
EML4	-0.3095926	7.10264734	12.3316682	0.00044534	0.03670924
BCL11B	-0.4178802	6.64325291	12.313791	0.00044962	0.03670924
SOCS3	0.98607135	3.97707589	12.2950229	0.00045417	0.03670924
ELK4	-0.2175191	8.69192668	12.2944318	0.00045431	0.03670924
AC068491.1	0.87903983	4.87604267	12.2523985	0.00046466	0.03719455
RGS2	0.4603161	7.9733602	12.1987179	0.00047822	0.0379258
IGFBP5	-0.5021814	8.65089126	12.0953528	0.00050548	0.03971932
EEF2	-0.252998	11.4295206	12.0643968	0.00051394	0.04001719
ATF6B	-0.5703589	4.26298272	12.0401414	0.00052067	0.04017604
OSBPL10	-0.5615798	5.26676972	11.970873	0.00054039	0.04132507
AC114752.3	1.81244367	3.89896497	11.8805856	0.00056722	0.04299335
MRVI1	0.61302784	5.03574788	11.8287785	0.00058322	0.04381842
POLR2G	0.77108771	4.0326349	11.8082945	0.00058967	0.04391791
PAX5	-0.4707259	7.60718085	11.7744051	0.00060051	0.04433916
CTSG	1.2488279	3.70017434	11.6032009	0.00065838	0.04799988
GUCY1B3	0.82847249	6.54272711	11.5949985	0.00066129	0.04799988
SLC9A3R2	-0.4065034	9.80548115	11.5456631	0.00067907	0.04887626
FCER1G	0.54743539	6.39330586	11.5220892	0.00068774	0.04908748
CD9	1.19311157	4.97250705	11.4867666	0.00070093	0.04961575

Supplementary file 5: Details of affected pregnancies. Abbreviations: MA, maternal age; GA, gestational age; Fraction, fetal RNA fraction; ART, assisted reproduction technology; <p10; intra-uterine growth retardation; EarlyPE, early-onset pre-eclampsia; LatePE, late-onset pre-eclampsia; PHT, pre-existent hypertension; PIH; pregnancy-induced hypertension; SPB, spontaneous preterm birth; EOPE_CRF, early-onset pre-eclampsia superimposed on chronic renal failure.

ID	Hospital	Origin	Group	MA	GA	Fraction	ART	Partus_wk	Partus_day	Fetal_sex	pvalue	<p10	EarlyPE	LatePE	PHT
P01-0035	VUMC	Fetal	IUGR	35	74	6.64	Yes	39	0	Boy	p5	Yes	No	No	No
P01-0169	VUMC	Fetal	IUGR	36	70	7.55	Yes	40	2	Boy	p5	Yes	No	No	No
P01-0204	VUMC	Fetal	IUGR	40	75	5.3	No	41	4	Girl	p3	Yes	No	No	No
P01-0222	VUMC	Fetal	IUGR	36	71	3.24	Yes	39	6	Girl	p5	Yes	No	No	No
P01-0446	VUMC	Fetal	IUGR	38	74	13.21	Yes	37	3	Girl	p5	Yes	No	No	No
P01-0447	VUMC	Fetal	IUGR	39	80	5.52	No	39	6	Boy	p10	Yes	No	No	No
P02-0053	AMC	Fetal	IUGR	33	88	3.79	No	38	4	Boy	p5	Yes	No	No	No
P02-0087	AMC	Fetal	IUGR	30	74	7.88	No	39	6	Boy	p5	Yes	No	No	No
P02-0117	AMC	Fetal	IUGR	40	71	13.98	Yes	39	4	Girl	p3	Yes	No	No	No
P02-0397	AMC	Fetal	IUGR	36	91	5.7	No	40	3	Boy	p10	Yes			
P02-0144	AMC	Maternal	LOPE	36	77	6.7	No	38	5	Girl	p10-50	No	No	PE	No
P02-0257	AMC	Maternal	LOPE	23	94	7.79	No	37	4	Boy	p10-50	No	No	PE	No
P01-0064	VUMC	Maternal	PHT	35	73	7.96	No	38	4	Girl	p50-90	No	No	No	Yes
P01-0266	VUMC	Maternal	PHT	34	85	11.11	Yes	38	4	Girl	p50-90	No	No	No	Yes
P01-0293	VUMC	Maternal	PHT	41	73	8.49	Yes	40	4	Girl	p10-50	No	No	No	Yes
P02-0029	AMC	Maternal	PHT	42	73	9.48	No	28	1	Girl	p10-50	No	No	No	Yes
P01-0230	VUMC	Maternal	PIH	32	80	9.69	No	41	0	Girl	p95	No	No	PIH	No
P02-0055	AMC	Maternal	PIH	40	98	14.35	No	41	4	Girl	p50-90	No	No	PIH	No
P02-0070	AMC	Maternal	PIH	42	76	9.73	No	39	6	Girl	p50	No	No	PIH	No
P01-0060	VUMC	Maternal	PIH_IUGR	39	88	6.54	No	36	5	Girl	<p2.3	Yes	No	PIH	No
P01-0032	VUMC	Mixed	SPB	42	70	5.27	Yes	34	5	Girl	p10-50	No	No	No	No
P01-0042	VUMC	Mixed	SPB	39	90	14.56	Yes	32	6	Boy	p10-50	No	No	No	No
P01-0080	VUMC	Mixed	SPB	37	72	4.39	Yes	36	6	Girl	p50-90	No	No	No	No
P01-0201	VUMC	Mixed	SPB	40	85	4.23	Yes	36	6	Boy	p50	No	No	No	No
P01-0229	VUMC	Mixed	SPB	34	70	4.19	Yes	30	0	Boy	p50-90	No	No	No	
P01-0232	VUMC	Mixed	SPB	33	72	5.63	No	35	5	Boy	p50	No	No	No	No
P01-0435	VUMC	Mixed	SPB	32	77	7.46	Yes	36	5	Girl	p10-50	No	No	No	No
P02-0002	AMC	Mixed	SPB	35	87	8.74	No	31	3	Girl	p50	No	No	No	No
P02-0014	AMC	Mixed	SPB	34	85	6.97	No	34	3	Girl	p10-50	No	No	No	No
P02-0017	AMC	Mixed	SPB	30	94	18.26	No	33	2	Boy	p50-90	No	No	No	No
P02-0095	AMC	Mixed	SPB	28	88	4.47	No	33	0	Boy	p50	No	No	No	No
P02-0176	AMC	Mixed	SPB	36	88	10.76	No	35	5	Girl	p10-50	No	No	No	No
P02-0251	AMC	Mixed	SPB	38	70	11.28	No	31	5	Girl	p10-50	No	No	No	No
P01-0278	VUMC	Mixed	EOPE_CRF	36	72	4.32	No	34	5	Boy	p10-50	No	PE	No	No

Supplementary file 6: Principle of the data-driven supervised self-learning SVM algorithm for biomarker identification. To demonstrate the principle of the variant of the leave-one-out cross-validation (LOOCV) analysis we applied, consider the presence of two different sample sets with the major change in differential gene signature expected to be either fetal (i.e. placental) in origin or non-fetal (i.e. maternal) in origin. For the purpose of clarity, we use two pregnant samples as representative for the former (fetal origin) and two non-pregnant samples for the latter (maternal origin). Using the data set from the normal reference cohort and with the optimal bioinformatic settings as described in the manuscript text, these samples are tested likewise in two separate comparisons. In practice this is done by marking these samples differently in the column 'group' in the 'sample info' file used in the thromboSeq.R script (test samples: X, pregnant samples: P, non-pregnant samples: NP). The outcome (heatmaps) of the 3-way comparisons with a 3-way ANOVA are shown in 6A and 6B with patterns as expected. The samples with fetal origin (pregnant) (green) segregate with the pregnant samples (blue) (6A), the samples with maternal origin (non-pregnant) (green) segregate with the non-pregnant controls (red). By analogy, for samples from affected pregnancies, individual samples can be added likewise. When segregrating as a distinct subgroup, additional samples from affected pregnancies with similar patterns can be combined to assure statistical signifance. For all comparisons, the three criteria set should be met: i. Pregnant versus non-pregnant discrimination should remain completely intact as visualized in the corresponding heatmaps. ii. The group t-test should remain to show the highest significance; and iii. The affected pregnancies should cluster as unique subgroups within the pregnancy group (discrete arms in the heatmap dendrogram).

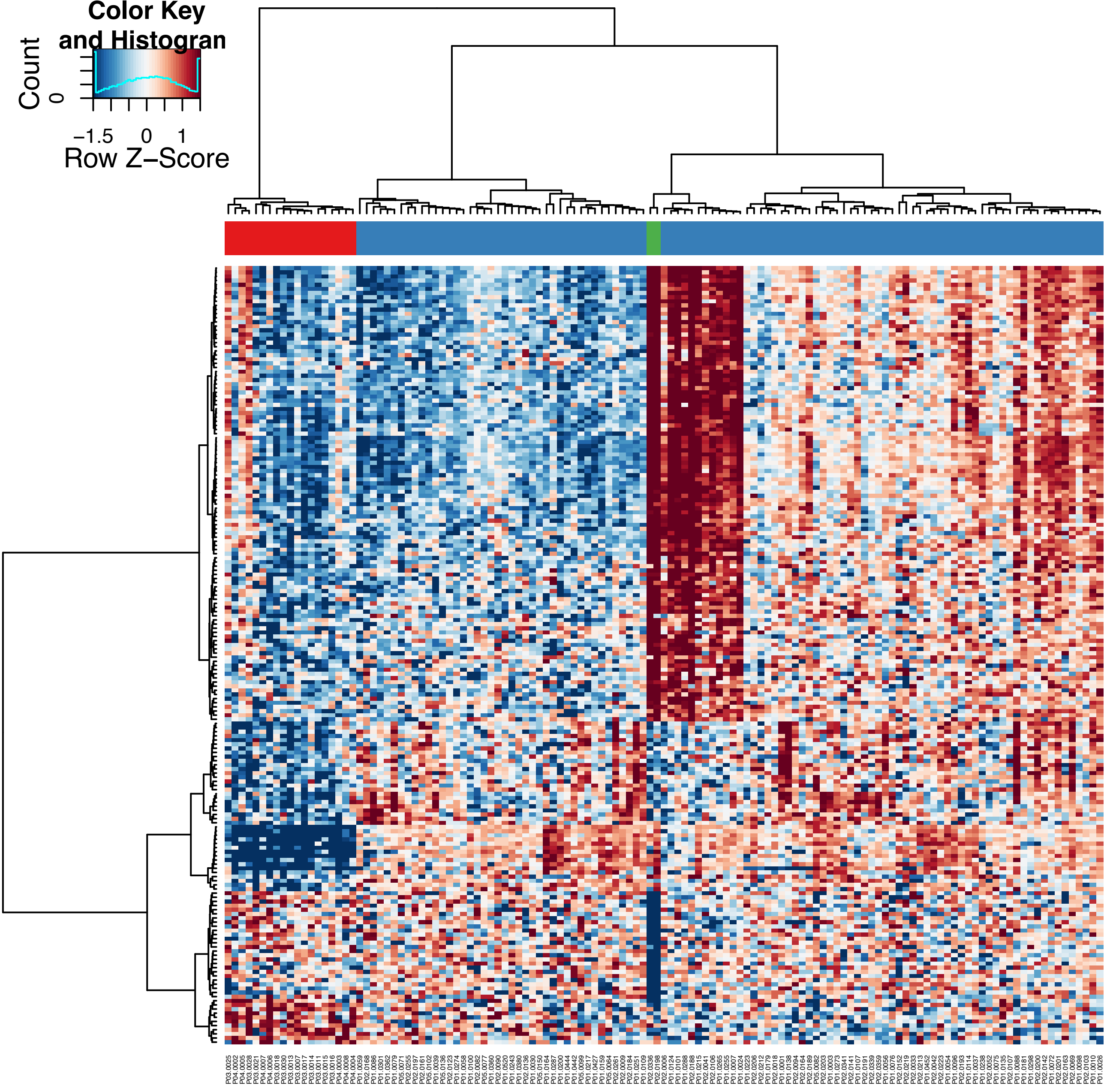
Color Key
and Histogran
Count
0
−1.5
0
1
Row Z−Score

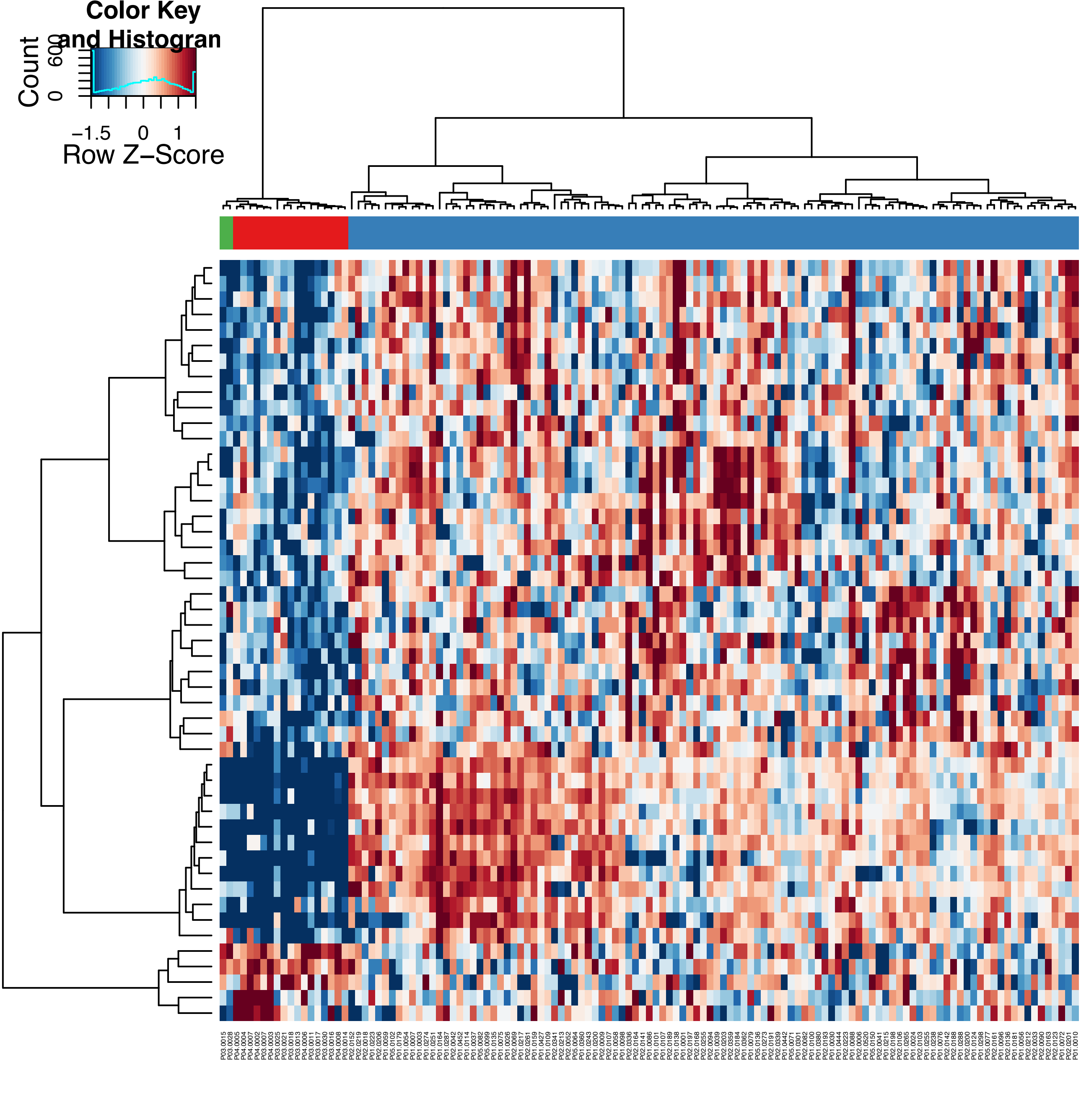
Color Key
and Histogran
Count
0
600
−1.5
0
1
Row Z−Score

Supplementary File 7: Early-onset pre-eclampsia superimposed on chronic renal failure. The EOPE samples segrates as a distinct subgroup (green) in combination with a single *non-pregnant* control sample. Normal pregnant samples: blue, non-pregnant samples: red.

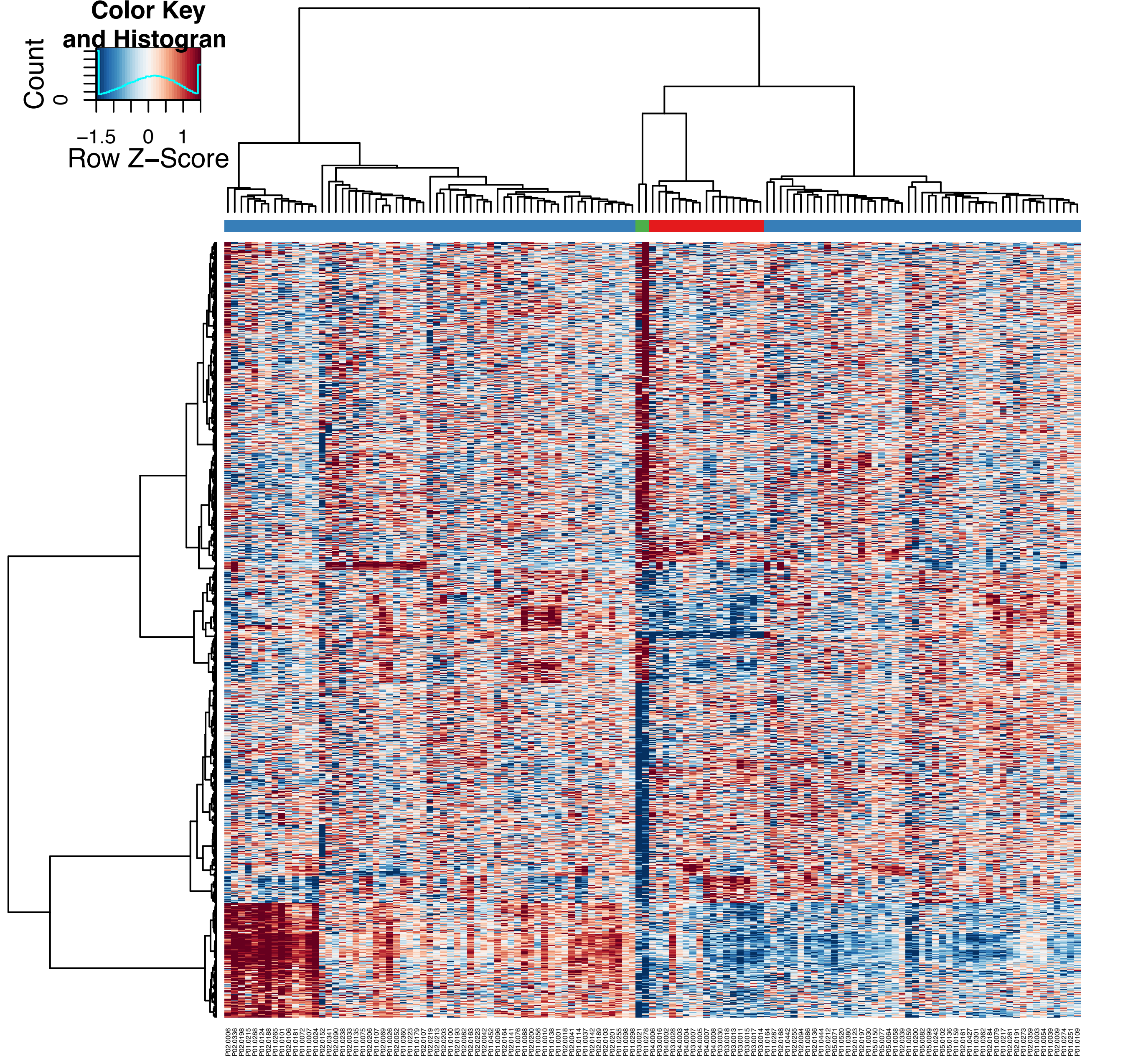
Color Key
and Histogran
Count
0
−1.5
0
1
Row Z−Score

Supplementary File 8: Details of samples from independent first trimester plasma RNA cohort used for validation by RT-qPCR of biomarker candidates. Clinical diagnosis was independently confirmed by one of the co-authors (MdB). Lab #: laboratory number; GA: gestational age in days; MA: maternal age in years.

#	Clinical diagnosis	Remarks	Lab #	GA	MA
1	HELLP syndrome		89	85	33
2	Intra-uterine growth retardation		747	83	34
3	Intra-uterine growth retardation		2067	96	36
4	Intra-uterine growth retardation		2427	71	30
5	Intra-uterine growth retardation	Intra-uterine fetal death week 39	590	96	35
6	Intra-uterine growth retardation		139	70	31
7	Intra-uterine growth retardation	Pregnancy-induced hypertension	1917	91	31
8	Intra-uterine growth retardation	Prematurity	564	154	38
9	Pre-eclampsia	Symptoms week 34	312	88	36
10	Pregnancy-induced hypertension		2429	95	38
11	Pregnancy-induced hypertension		258	80	36
12	Pregnancy-induced hypertension		333	72	35
13	Normal pregnancy		496	79	31
14	Normal pregnancy		515	89	29
15	Normal pregnancy		897	81	33
16	Normal pregnancy		1081	79	32
17	Normal pregnancy		1039	75	33
18	Normal pregnancy		1499	84	30
19	Non-pregnant		34	0	41
20	Non-pregnant		35	0	25
21	Non-pregnant		36	0	40
22	Non-pregnant		37	0	36
23	Non-pregnant		38	0	40
24	Non-pregnant		39	0	41

Supplementary File 9: Validation of *PRKG1* and *AVPR1A* by RT-qPCR in an independent first trimester plasma cohort.

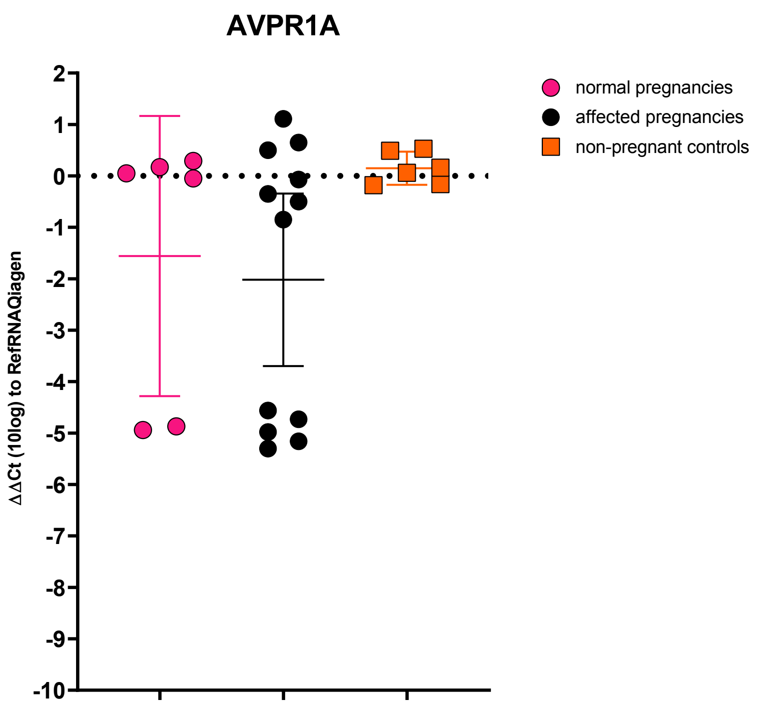

AVPR1A
normal pregnancies
affected pregnancies
non-pregnant controls
ΔΔCt (10log) to RefRNAQiagen
2
1
0
-1
-2
-3
-4
-5
-6
-7
-8
-9
-10

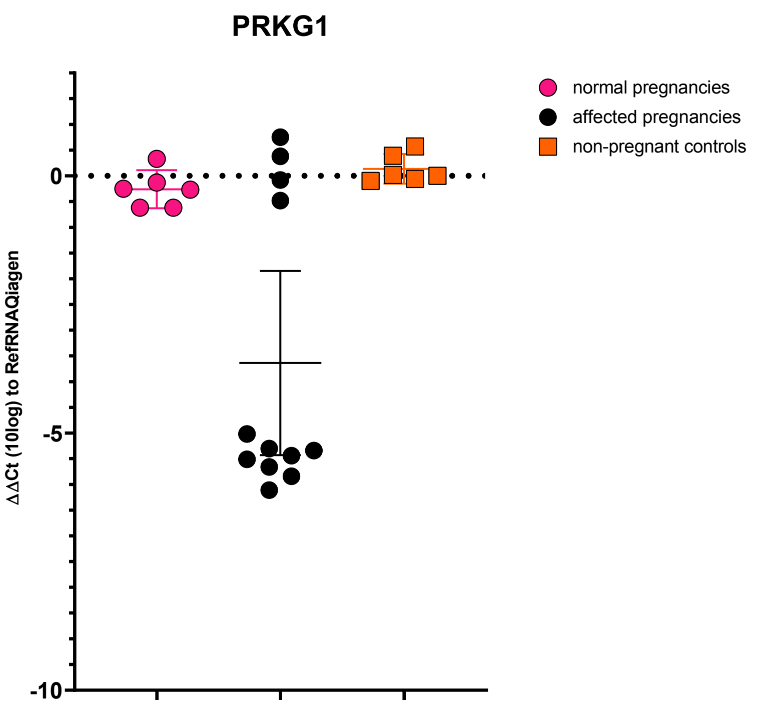

PRKG1
normal pregnancies
affected pregnancies
non-pregnant controls
ΔΔCt (10log) to RefRNAQiagen
0
-5
-10

Supplementary File 10: Significance of NRIP1 levels in pregnancies complicated by pre-eclampsia or trisomy 21. The data correspond to the samples tested in Figure 5.

Comparison relative expression NRIP1

	Normal pregnancy versus (p-value)	Non-pregnant versus (p-value)
Normal (n=6)		0.101
IUGR	0.9995	0.0416
PE	<0.0001	0.0015
PIH	0.9889	0.5821
T21	0.0006	0.1563

Tukey's multiple comparisons test	Mean Diff.	95.00% CI of diff.	Significant?	Summary	Adjusted P Value
normal pregnancies vs. non-pregnant controls	-0.5017	-1.066 to 0.06222	No	ns	0.101
normal pregnancies vs. IUGR pregnancies	0.05667	-0.4867 to 0.6000	No	ns	0.9995
normal pregnancies vs. PE pregnancies	-1.53	-2.221 to -0.8394	Yes	****	<0.0001
normal pregnancies vs. PIH pregnancies	-0.1367	-0.8273 to 0.5540	No	ns	0.9889
normal pregnancies vs. T21 pregnancies	-1.016	-1.646 to -0.3854	Yes	***	0.0006
non-pregnant controls vs. IUGR pregnancies	0.5583	0.01496 to 1.102	Yes	*	0.0416
non-pregnant controls vs. PE pregnancies	-1.028	-1.719 to -0.3377	Yes	**	0.0015
non-pregnant controls vs. PIH pregnancies	0.365	-0.3256 to 1.056	No	ns	0.5821
non-pregnant controls vs. T21 pregnancies	-0.5142	-1.145 to 0.1163	No	ns	0.1563
IUGR pregnancies vs. PE pregnancies	-1.587	-2.261 to -0.9127	Yes	****	<0.0001
IUGR pregnancies vs. PIH pregnancies	-0.1933	-0.8673 to 0.4806	No	ns	0.9451
IUGR pregnancies vs. T21 pregnancies	-1.073	-1.685 to -0.4603	Yes	***	0.0002
PE pregnancies vs. PIH pregnancies	1.393	0.5959 to 2.191	Yes	***	0.0002
PE pregnancies vs. T21 pregnancies	0.5142	-0.2318 to 1.260	No	ns	0.3033
PIH pregnancies vs. T21 pregnancies	-0.8792	-1.625 to -0.1332	Yes	*	0.0146

Supplementary file 11: Quantitative analysis of linear and circular *NRIP*1 RNA in pregnancies complicated by pre-eclampsia and pregnancies with a trisomy 21 fetus. circ*MAN1A2* was used as housekeeping gene, reference RNA was used as calibrator. Pre-eclamptic pregnancies showed an increase in linear *NRIP1* compared to circular *NRIP1* while trisomy 21 pregnancies do not.

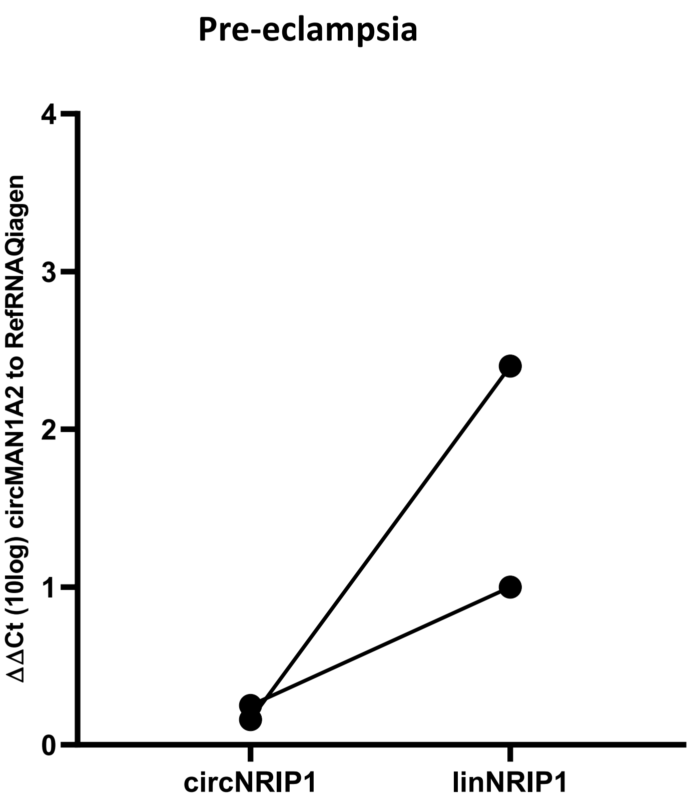

Pre-eclampsia
ΔΔCt (10log) circMAN1A2 to RefRNAQiagen
0
1
2
3
4
circNRIP1
linNRIP1

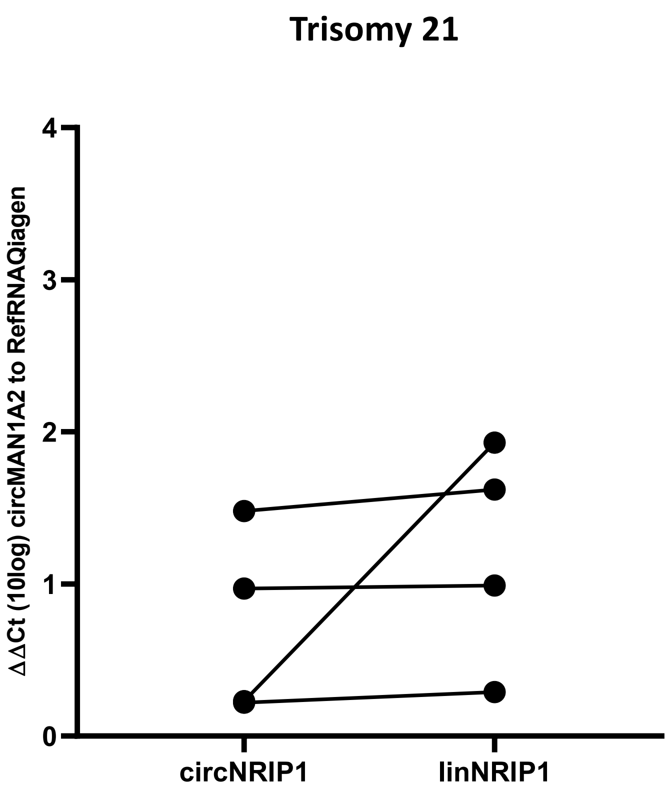

Trisomy 21
ΔΔCt (10log) circMAN1A2 to RefRNAQiagen
0
1
2
3
4
circNRIP1
linNRIP1
